# Leveraging Community Partnerships to Craft Minnesota’s Multisector Blueprint on Aging

**DOI:** 10.1093/geroni/igaf122.1112

**Published:** 2025-12-31

**Authors:** Farah Baig, Rajean Moone

**Affiliations:** Age-Friendly Minnesota Council, St. Paul, Minnesota, United States; University of Minnesota, Minneapolis, Minnesota, United States

## Abstract

In 2019, the Governor of Minnesota established the Age-Friendly Council to unite stakeholders from state agencies, academia, tribal organizations, and the public. The Council’s goals were to enroll the state in the WHO’s Network of Age-Friendly Communities and to guide the creation of a Multisector Blueprint for Aging. This paper examines the community-based participatory research methods used to develop the Blueprint, which engaged over 3,500 individuals statewide in meaningful discussions. Qualitative analysis of these conversations identified four key domains: Connected Communities, Emergency Preparedness, Individual Rights and Safety, Optimized Health and Longevity, and Economic Security and Vitality. The paper also explores the collaboration between the University of Minnesota’s Center for Healthy Aging and Innovation, the Geriatric Workforce Enhancement Program, and public sector partners in advancing age-friendly initiatives. Additionally, it highlights strategies for engaging diverse geographic and cultural communities through action teams that define priorities, establish measurable outcomes, and implement effective strategies. By integrating voices from multiple sectors and communities through participatory methods, Minnesota’s aging initiatives foster innovation and long-term sustainability. The Blueprint serves as a model for other states seeking to create inclusive environments where older adults can thrive through coordinated, evidence-based policies and programs.

